# Analysis of the Radial Force of a Piezoelectric Actuator with Interdigitated Spiral Electrodes

**DOI:** 10.3390/mi15111378

**Published:** 2024-11-15

**Authors:** Yateng Wang, Tianxing Ren, Yuan Ren, Ruijie Gu, Yonggang Liu

**Affiliations:** School of Mechatronics Engineering, Henan University of Science and Technology, Luoyang 471003, China; m18338856199@163.com (Y.W.); 15637909901@wo.cn (T.R.); rtx0225@hotmail.com (Y.R.); jackgu0214@163.com (R.G.)

**Keywords:** piezoelectric actuator, interdigital spiral electrode, radial force, electric signal

## Abstract

The actuator is a critical component of the micromanipulator. By utilizing the properties of expansion and contraction, the piezoelectric actuator enables the manipulator to handle and grasp miniature objects during micromanipulation. However, in piezoelectric ceramic disc actuators with conventional surface electrode configurations, the actuating force generated in the radial direction is relatively limited. When used as the actuation element of the manipulator, achieving regulation over a wide range of operating strokes becomes challenging. Therefore, altering the electrode structure is necessary to generate a greater radial force, thus enhancing the positioning and grasping capabilities of the operating arm. This paper investigates a piezoelectric actuator with interdigitated spiral electrodes, featuring a constant pitch between adjacent electrodes. The radial force was tested under mechanical clamping conditions, and the influence of the electrical signal was examined. The characteristics of the electrode structure were described, and the working principles of the piezoelectric actuators were analyzed. Theoretical equations were derived for the macroscopic characterization of the radial clamping force of the actuator, based on the piezoelectric constitutive equation, geometric principles, and Bond matrix transformation relationships. A finite element model was developed, reflecting the features of the electrode structure, and finite element simulations were employed to verify the theoretical equations for radial force. To prepare the samples, encircled interdigitated spiral electrode lines were printed on the PZT-52 piezoelectric ceramic disc using a screen printing method. The clamping force experimental platform was established, and experiments on the clamping radial force were conducted with electrical signals of varying waveforms, frequencies, and voltages. The experimental results show that the piezoelectric ceramic disc actuator with an interdigitated spiral electrode line structure, when excited by a stable sine wave operating at 200 V and 0.2 Hz, generated a peak force of 0.37 N. It was 1.76 times greater than that produced by a previously utilized piezoelectric disc with conventional electrode structures.

## 1. Introduction

Piezoelectricity is proposed as a solution for a low-power, fast-switching, and high-force micro-actuator, among alternatives based on thermal, magnetic, and electrostatic driving principles [1]. Piezoelectric actuators utilize the inverse piezoelectric effect to convert micro-scale deformations into macroscopic output forces through electromechanical coupling, thereby generating actuation performance [2]. They offer advantages such as compact size, simple structure, fast response speed, high-precision positioning, and strong resistance to electromagnetic interference [3,4]. As a result, they have been widely employed in the actuation and regulation of advanced micro-machinery.

Piezoelectric discs were used as actuators, with positive and negative electrodes positioned on the upper and lower surfaces, respectively. These conventional actuators utilize the transverse piezoelectric effect. The *d*_31_ piezoelectric coefficient induces deformation of the disc when the circular edges are clamped [5]. While *d*_31_ is a relatively small transverse piezoelectric constant, the resulting macroscopic output displacement is insufficient, limiting practical applications. A growing body of research has focused on substituting surface electrodes with interdigital electrodes due to their superior longitudinal piezoelectric constant, *d*_33_. N. Chidambaram and A. Mazzalai experimentally demonstrated that the performance of piezoelectric actuators operating in the *d*_33_ mode showed a 20% improvement compared to those functioning in the *d*_31_ mode [6,7]. As a result, piezoelectric actuators with interdigital electrodes, utilizing the longitudinal piezoelectric constant *d*_33_, have attracted significant interest among designers [8].

Liu Yonggang et al. [9] indicated that the strain of piezoelectric actuators with interdigital electrode structures was greater than that of traditional surface electrode actuators. They also investigated a piezoelectric actuator with a locally annular interdigital electrode structure arranged on a piezoelectric circular ceramic disc. By setting up a displacement testing platform, they measured the radial displacement generated by the actuator. The results showed that the peak radial displacement of the actuator was 2.5 times greater than that of surface electrode piezoelectric actuators with the same structure [10]. Cuong H. Nguyen et al. [11] studied the influence of different electrode structures on the bending performance of piezoelectric micro-actuators and applied them to the lenses of automatic focusing cameras. They compared the performance of micro-actuators under equal electric field intensity conditions using the finite element method. The results demonstrated that the driving performance of the interdigital electrode structure was superior to that of the traditional surface electrode.

The research primarily focused on the strain, displacement, and practical applications of interdigital piezoelectric actuators. However, the output force of the actuator has not been tested. In experimental testing, the internal electric domain distribution of the piezoelectric actuator in a mechanically clamped state undergoes certain changes, so the properties observed under displacement output conditions are not fully applicable to actuation output conditions.

Researchers have conducted theoretical studies on the output force characteristics of piezoelectric ceramic actuators. Crawley and Luis [12] examined the actuating force and moment formulas, considering the shear deformation of the adhesive layer in piezoelectric ceramic actuation. They provided a distribution curve of the actuation force for piezoelectric ceramics, which effectively simulated the driving performance of piezoelectric materials. Later, Crawley and Anderson [13] developed the actuation formula for piezoelectric materials, taking bending deformation into account but neglecting shear deformation. Sonti et al. [14] studied piezoelectric actuators with different shapes and uniform polarization and analyzed their equivalent output actuation force. They also investigated the influence of actuator shape on the modal response of plate structures and derived a general expression for the equivalent actuation force of piezoelectric actuators based on small deflection theory. Deraemaeker et al. [15] derived the analytical expression for the equivalent force of orthotropic piezoelectric actuators under general conditions, using Hamilton’s principle and two different mathematical methods based on small deflection theory. Both methods yielded the same analytical expression for the equivalent force. These force output characteristics are theoretical expressions derived by considering various deformation factors of the bonding layer in piezoelectric materials. No experimental tests or analyses have been conducted on these piezoelectric actuators bonded to laminated structures. In particular, when piezoelectric actuators are used as the core component of microgrippers to handle and manipulate micro-parts, studying the variation in radial force with the driving voltage signal is of great significance for the development of micro-clamps [16]. This is because most microgrippers utilize piezoelectric stacking actuators to generate input force [17,18,19,20,21]. The input force of the microgripper is equal to the sum of the axial forces generated by individual piezoelectric elements within the piezoelectric stack. This is comparable to a single helical interdigitated electrode piezo disc actuator, which is capable of generating the same amount of radial force. However, due to structural limitations, piezoelectric stacks are difficult to integrate into a large inertia block, thereby limiting their driving capacity [4,22,23]. To increase driving capacity, multiple piezoelectric stacks are typically required, which increases the cost of the driver. For these reasons, piezoelectric actuators with low driving costs have become a focal area of research [24,25].

Therefore, this study investigates the radial force of a piezoelectric ceramic disc actuator with interdigitated spiral electrodes. This approach offers the potential to replace piezoelectric stacked actuators with piezoelectric actuators featuring interdigitated electrode structures in micro-operation applications, such as microgrippers. Additionally, the radial actuation force was derived using the piezoelectric constitutive equation, and the stress distribution of the actuator in the clamped state was analyzed using the finite element method. Radial force experiments were conducted to explore the effect of the applied electrical signal on the radial output clamping force of the piezoelectric actuator. This research provides a reference for the performance evaluation of external load driving mechanisms in actuators.

## 2. Structural Characteristics and Driving Principles

**Figure 1:**
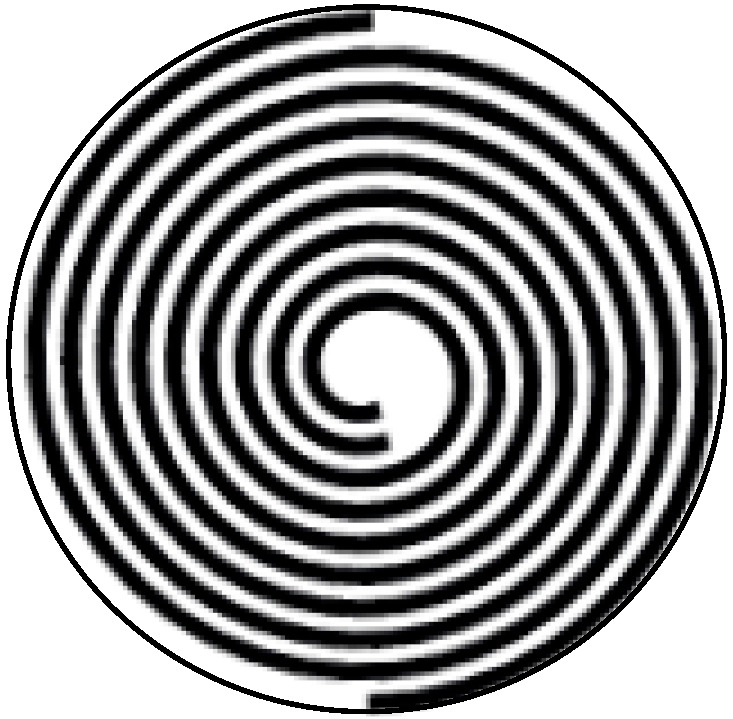
Structure of the piezoelectric actuator with spiral electrodes.

Figure 1 shows a piezoelectric actuator with equidistant spiral electrodes. Interdigital electrodes are used in the piezoelectric disc actuator. This configuration allows electrodes to be printed on both the upper and lower surfaces of the disc, thereby enhancing the output force. A pair of opposite electrodes is arranged on the upper surface, while the other pair is placed on the lower surface. Within the disc plane, the positive and negative electrode lines are arranged in a spiral encircling pattern, with symmetrical electrode lines on both the upper and lower surfaces. The electrode lines conform to an Archimedean spiral trajectory, characterized by the radius at any given point on the line being a function of the angle of rotation about the central axis. The two electrode lines in the rotating plane expand equally with each rotation cycle, maintaining a constant distance between them.

A piezoelectric disc undergoes isotropic deformation upon the application of an electric signal, due to the transverse piezoelectric effect of the piezoelectric ceramics. Polarization occurs along the axial direction [26]. In addition to functioning as the working electrode, the spiral electrode lines also serve as the polarized electors of the actuator [27]. The polarization direction of the main axis 3 is radial. In response to an external electric field, the actuator produces radial stretching and shrinking deformation through the longitudinal piezoelectric effect. The piezoelectric constants also vary with the polarization direction [28]. By utilizing the longitudinal piezoelectric constant *d*_33_, which is significantly larger than the transverse piezoelectric constant *d*_31_, the piezoelectric actuator with spiral electrodes of equal spacing can substantially improve performance [29].

**Figure 2:**
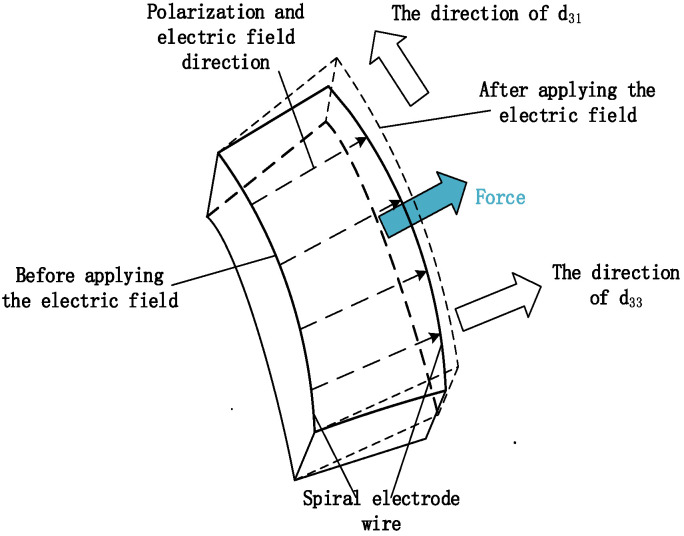
The deformation diagram of the electrode spacing sub-body.

To elucidate the driving principle of the spiral electrode actuator, a portion of the electrode spacing sub-body is abstracted along the length direction of the spiral electrode line. It is assumed that a small sub-body is composed of consistent material and uniformly polarized within the electrode gap sub-body. Electric field lines are uniformly distributed from the positive to the negative electrodes along the polarization axis 3. The electric field is parallel to the surface of the disc. The actuator responds to the electric field by expanding and contracting along the polarization main axis 3, with the deformation being related to the piezoelectric constant *d*_33_. Deformation in the two directions perpendicular to the main axis 3 depends on the piezoelectric constant *d*_31_ [30]. The deformation of the interelectrode spacing sub-bodies results from the combination of both deformation effects. This deformation generates stress at the interfaces due to the interaction between crystal grains. Within the actuator, a finite number of similar electrode gap sub-bodies can be identified, with the deformation and stress of each sub-body being superimposed to form the overall deformation and stress of the actuator. Figure 2 illustrates a schematic diagram of the generated deformation and driving force.

## 3. Radial Force Analysis

The mechanical and electrical characteristics of the piezoelectric effect can be treated as independent variables, allowing for the derivation of different piezoelectric equations depending on the chosen independent variables [31]. In the presence of an external electric field, the deformation and stress of equidistant spiral electrode piezoelectric actuators can primarily be described by the equation representing the inverse piezoelectric effect. When analyzing the deformation, it is assumed that the element is mechanically free. In the strain analysis, stress and electric field are considered independent variables. The equation representing the inverse piezoelectric effect is provided as follows:

(1) $$\varepsilon =S^{E}\sigma +dE,$$

The stress in an element under a mechanically clamped state is described by treating strain and electric field strength as independent variables. The equation representing the inverse piezoelectric effect is given as follows:

(2) $$s=C^{E}e-eE,$$

where ε is radial strain; σ represents stress; $S^{E}$ is the elastic compliance coefficient; E is radial electric field intensity; $C^{E}$ is the elastic stiffness coefficient; e is piezoelectric stress coefficient.

In this study, a clamped–clamped mechanical boundary condition is considered for testing the radial force. Based on the operating conditions, the environment of the actuator, and the relevant literature [32,33,34], the piezoelectric constitutive equation corresponding to the second boundary condition for piezoelectric materials is adopted. This condition involves mechanical clamping and short-circuiting, as represented by Equation (2). For a spiral electrode disc piezoelectric actuator, only radial extension and contraction are considered. Assuming continuity of the internal stress within the component, the strain ε is set to zero to derive the expression for the internal stress of the actuator when it is fixed and clamped, as is the case during testing. Upon application of an external electric signal, the relationship between stress and electric field can be expressed in matrix form as follows:

(3) $$\left[\begin{array}{l} \sigma_{1}\\ \sigma_{2}\\ \sigma_{3}\\ \tau_{yz}\\ \tau_{zx}\\ \tau_{xy} \end{array}\right]=\left[\begin{array}{ccc} \begin{array}{l} e_{11}\\ e_{12}\\ e_{13}\\ e_{14}\\ e_{15}\\ e_{16} \end{array} & \begin{array}{l} e_{21}\\ e_{22}\\ e_{23}\\ e_{24}\\ e_{25}\\ e_{26} \end{array} & \begin{array}{l} e_{31}\\ e_{32}\\ e_{33}\\ e_{34}\\ e_{35}\\ e_{36} \end{array} \end{array}\right]•\left[\begin{array}{l} E_{1}\\ E_{2}\\ E_{3} \end{array}\right]$$

Based on the symmetry of piezoelectric materials, after polarization in the radial direction, the relationship between stress and electric field intensity can be expressed as follows:

(4) $$\left[\begin{array}{l} \sigma_{1}\\ \sigma_{2}\\ \sigma_{3}\\ \tau_{yz}\\ \tau_{zx}\\ \tau_{xy} \end{array}\right]=\left[\begin{array}{ccc} \begin{array}{l} 0\\ 0\\ 0\\ 0\\ e_{15}\\ 0 \end{array} & \begin{array}{l} 0\\ 0\\ 0\\ e_{15}\\ 0\\ 0 \end{array} & \begin{array}{l} e_{31}\\ e_{31}\\ e_{33}\\ 0\\ 0\\ 0 \end{array} \end{array}\right]•\left[\begin{array}{l} E_{1}\\ E_{2}\\ E_{3} \end{array}\right]$$

Due to the unique structure of the interdigitated spiral electrodes, the electrodes also serve as the polarized electrodes. Consequently, the applied electric field is parallel to the direction of the polarization axis 3, with the electric field strength present only along axis 3 and zero in all other directions. Direction 3 is defined as the polarization direction of the material [34]. Equation (4) was simplified by reducing it to a two-dimensional plane for analysis, and rewritten as follows:

(5) $$\left[\begin{array}{l} \sigma_{3}\\ \sigma_{1}\\ \tau_{xy} \end{array}\right]=\left[\begin{array}{l} e_{33}\\ e_{31}\\ 0 \end{array}\right]•E_{3},$$

Through a rotational transformation, the physical spindle coordinate system *123* is converted into the *XYZ* coordinate system, which is used to describe and analyze the radial stress in piezoelectric elements. The matrix transformation relationship between stress and electric field can be expressed as follows:

(6) $$\begin{array}{l} \left\{\sigma \right\}=\left[M\right]\left\{\sigma^{\prime }\right\}\\ \left\{E\right\}=\left[Q\right]\left\{E^{\prime }\right\} \end{array},$$

where [*M*] and [*Q*] are coordinate transformation matrices.

By substituting Equation (6) into Equation (2), the stress induced by the applied external electric field, when the strain is zero, can be obtained [35].

(7) $$\left[\sigma^{\prime }\right]=-{\left[M\right]}^{-1}\left[e\right]\left[Q\right]\left[E^{\prime }\right]$$

The stress vector is represented as

(8) $$\left[\begin{array}{l} \sigma_{x}^{\prime }\\ \sigma_{y}^{\prime }\\ \tau_{xy} \end{array}\right]=-{\left[M\right]}^{-1}\left[e\right]E_{3}=-\left[\begin{array}{ccc} \cos^{2}\alpha & \sin^{2}\alpha & -2\sin\alpha \cos\alpha \\ \sin^{2}\alpha & \cos^{2}\alpha & 2\sin\alpha \cos\alpha \\ \sin\alpha \cos\alpha & -\sin\alpha \cos\alpha & \cos^{2}\alpha -\sin^{2}\alpha \end{array}\right]\left[\begin{array}{l} e_{33}\\ e_{31}\\ 0 \end{array}\right]•E$$

In the mechanical clamping state, the radial stress of the element is

(9) $$\sigma_{x}^{\prime }=-(\cos^{2}\alpha e_{33}+\sin^{2}\alpha e_{31})E_{3}$$

The radial force of actuator is

(10) $$F_{r}=2\sigma_{x}^{\prime }A_{r},$$

where *A_r_* is the radial contact area of the electrode region.

## 4. Finite Element Analysis

To eliminate the influence of internal stress concentration on the radial force test of piezoelectric elements, finite element analysis software ANSYS (Version 2019 R2, ANSYS, Inc., Canonsburg, PA, USA) was employed to analyze the stress distribution of the elements under clamping conditions, ensuring reliability. A disc with dimensions of 25 × 2 mm, a base radius of 1 mm for the electrode line, an electrode width of 0.6 mm, and an interelectrode distance of 0.6 mm were selected for the analysis. Both the positive and negative electrode helices of the actuator were designed with a maximum of nine turns.

Based on the driving principle analysis presented in Section 2 and the uniform field theory, we can make three assumptions: (1) The polarization direction is radial and perpendicular to the centerline of the electrodes. (2) The polarization directions of the elements on either side of the electrode centerline are opposite. (3) The elements are uniformly polarized, with fixed piezoelectric strain constants and stress constants.

**Figure 3:**
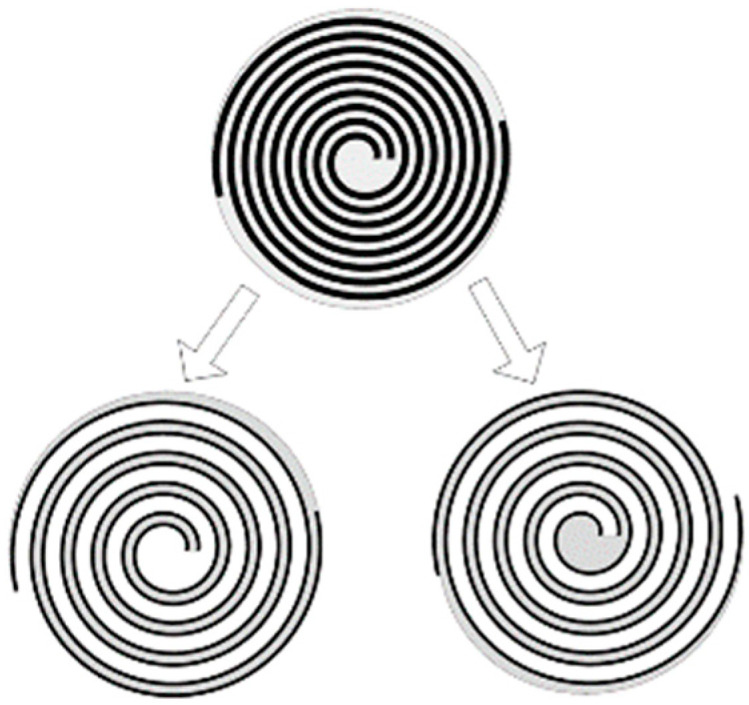
Overall model and segmentation models.

A three-dimensional model of the spiral electrode piezoelectric element was created using Creo software(Version 7.0.0.0, Creo Parametric, Inc., PTC, Boston, MA, USA). The model was then cut along the centerline of the spiral electrode. Both the complete model and the segmented models are shown in Figure 3. The final step involved importing the segmented models into ANSYS individually and combining them using Boolean operations.

The centerline of the spiral electrode is polarized in opposite directions on both sides. Therefore, it is necessary to establish two material models: Model 1, which is aligned with the positive direction of the element’s radial direction, and Model 2, which is aligned with the negative direction of the element’s radial direction. PZT-52 was selected as the material. The material parameters are provided in Table 1.

**Figure 4:**
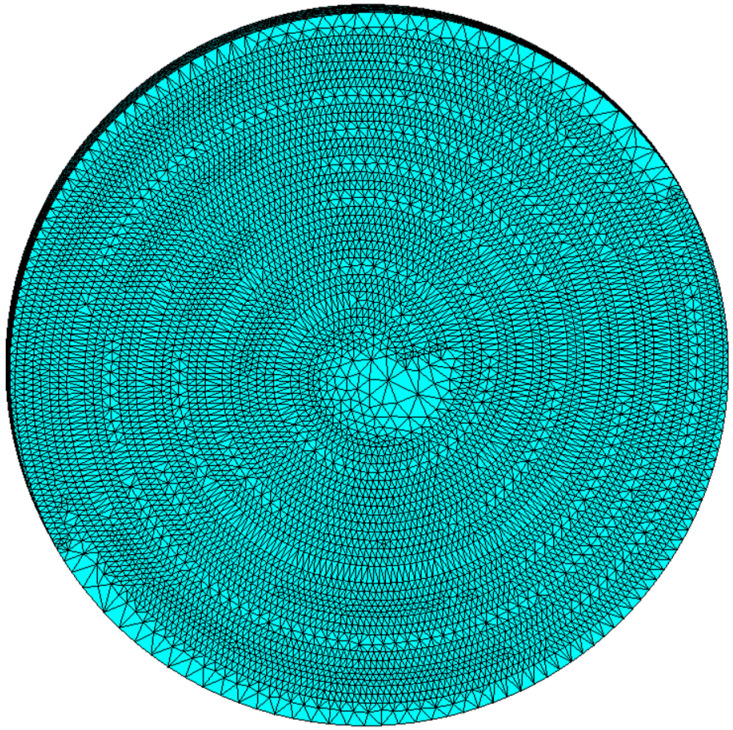
Diagram of the finite element meshing model.

The finite element mesh should be divided based on the material properties assigned to the corresponding models. Piezoelectric coupling analysis was performed using the SOLID98 element type. The mesh was generated using an intelligent division technique. The number of finite element elements is 330,000~560,000. Figure 4 shows the resulting mesh model.

**Figure 5:**
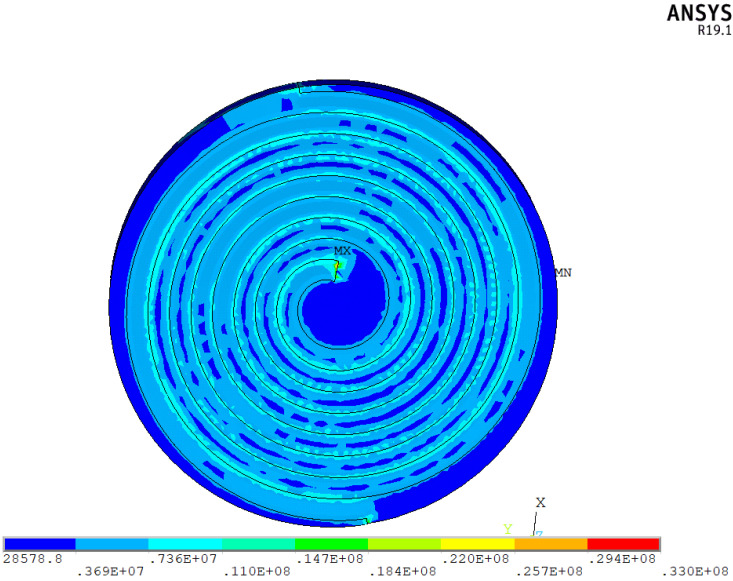
Radial stress distribution.

The fixed position of the actuator during the radial clamping force test was determined by the tangent of the outermost spiral electrode and the edge of the disc. Therefore, each nodal displacement at the edge of the piezoelectric element must be constrained in the stress finite element analysis of the spiral electrode piezoelectric actuator. The displacement boundary condition is applied to the 10° arc surface near the 270° position of the piezoelectric disc. The displacement constraint for all nodes is set as *S_i_* = 0 (i = *x*, *y*, *z*). The applied voltage load is as follows: for the Z = 0 plane, the positive electrode region is set to U = 200 V, and the negative electrode region is set to U = 0 V; for the Z = *h* surface (where *h* is the thickness of the piezoelectric disc), the positive electrode area is set to U = 200 V, and the negative electrode area is set to U = 0 V. Figure 5 shows the stress contour map resulting from the direct application of 200 V and 0 V to the positive and negative electrode regions of the disc.

From the distribution, it is evident that the radial stress at the clamping force detection point of the piezoelectric actuator reaches 3.69 × 10^6^ N/m^2^. There is a stress concentration at the starting point of the non-zero potential end of the spiral electrode line in the piezoelectric actuator, which amounts to 3.30 × 10^7^ N/m^2^. This stress concentration results from the specialized electrode structure. Within the piezoelectric elements, the minimum stress occurs at the edge of the non-mechanically clamped radial direction. Along both sides of the electrode line, there is a variation in the stress gradient across the entire element, ranging from the minimum stress to 7.36 × 10^6^ N/m^2^. The electrode line where a positive electric signal is applied exhibits a higher stress level. Consequently, the tangent point where the end of the positive spiral electrode wire meets the circumferential line of the piezoelectric ceramic disc should be selected as the detection point.

## 5. Preparation and Force Test

To conduct the radial clamping force test on the piezoelectric actuator with interdigitated spiral electrodes, several preparatory steps must be completed. Conductive silver paste was employed as the electrode material. The electrodes were printed on the piezoelectric ceramic disc substrate using screen printing. Prior to curing, the printed silver spiral electrodes must be heated in a box resistance furnace. The electrodes are symmetrically arranged on both the upper and lower surfaces of the ceramic disc [36]. In order to select piezoelectric samples suitable for polarization, the conductivity of the electrodes was tested using a multimeter.

**Figure 6:**
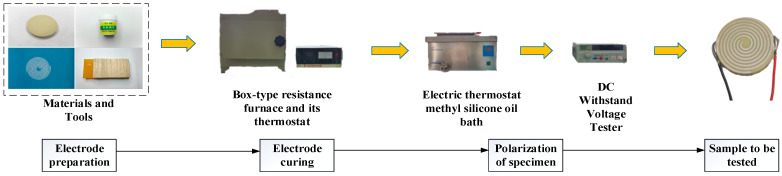
Flowchart for polarization and preparation of samples.

Before polarization, the piezoelectric sample contains an irregular arrangement of grain domains and does not exhibit piezoelectric properties. To enable the demonstration of the piezoelectric effect, polarization is necessary [37]. During the clamping force test, electrodes on the upper and lower surfaces of the piezoelectric sample were connected by soldering wires to facilitate polarization and load application. The specimens were polarized in an electric thermostatic oil bath containing methyl silicone oil. In this experiment, the piezoelectric samples were connected to a DC Withstand Voltage Tester via soldered wires. The voltage was gradually increased until it reached the required polarization voltage. An initial polarization temperature of 120 °C was maintained for approximately 60 min. After polarization, the samples were removed and placed in methyl silicone oil at room temperature to cool after the DC high voltage supply was disconnected. The samples were then cleaned with anhydrous alcohol and allowed to dry for 24 h. The preparation and polarization process is illustrated in Figure 6.

**Figure 7:**
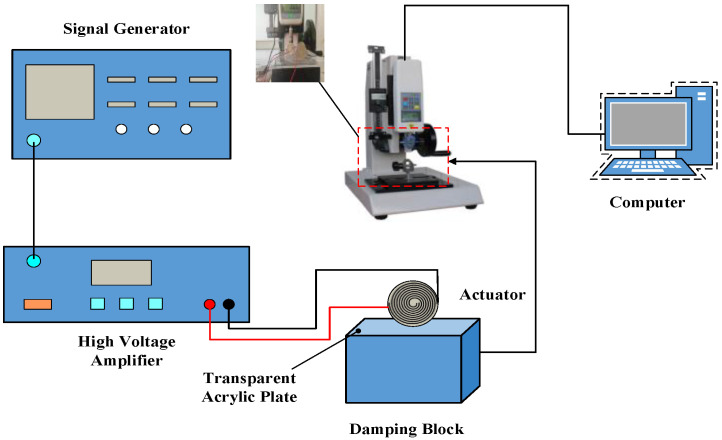
Schematic diagram of the force test.

To accurately control piezoelectric actuators, it is essential to examine the force characteristics of piezoelectric samples. This study investigates the effect of the electric signal on the clamping force of the actuator. A radial clamping force test system was designed to explore the relationship between the force exerted by piezoelectric actuators and the applied electrical signal. Figure 7 presents the schematic diagram of the force test.

**Figure 8:**
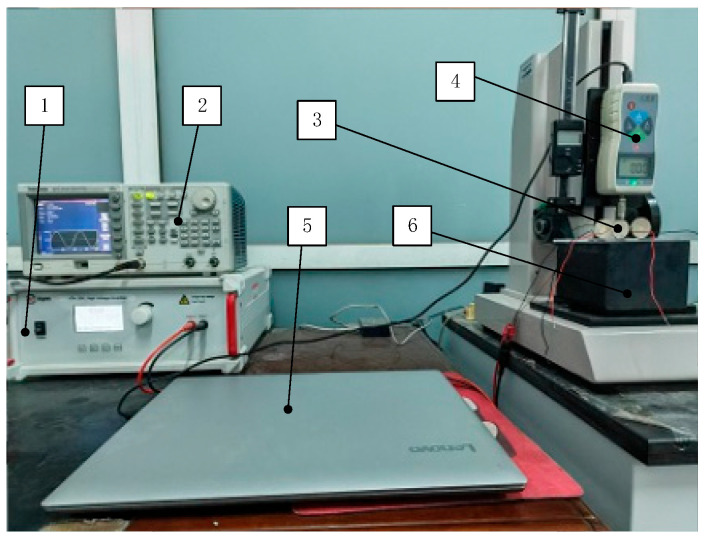
Clamping force experimental device: 1. Antai ATA-2021 High Voltage Amplifier (Aigtek Electronic Technology Ltd., Xian, China); 2. Tektronix AFG1022 Signal Generator (Tektronix, Inc., Johnston, IA, USA); 3. Samples; 4. Digital force gauge (YueQing Handpi Instruments Co., Ltd., Yueqing, China); 5. Computer; 6. Damping block.

An output signal parameter can be directly adjusted by the function generator, which generates the electrical signal waveform. The signal voltage is amplified by a high-voltage amplifier. By adjusting the amplification factor of the high-voltage amplifier, different voltages of the electric signal can be produced. Consequently, the electrical signal is applied to the piezoelectric actuator under test. During testing, actuators were mounted perpendicular to transparent acrylic plates along radial axes and were attached to a damping block. The damping block was positioned in the testing area of the digital force gauge. A combination of the test head and damping block was used to pre-tighten the piezoelectric samples. The data collected by the digital force gauge tester were processed by a computer. Figure 8 illustrates the clamping force experimental setup.

## 6. Analysis of Experimental Results

The clamping force was detected under various electrical signals using the experimental device through a single-factor variable method. In each test, the variable actuating the signal was changed every 30 s to ensure proper application of the electric signal to the piezoelectric actuator. This approach ensured the relative stability of the output data, allowing for the determination of the optimal value for the radial clamping force.

Due to multiple cycles of the excitation signal from the piezoelectric actuator, the repeatability of the response hysteresis curve deteriorated. This was evident in the response curve, which exhibited a rising hysteresis loop between the increasing and decreasing curves. Therefore, the experimental data represented the average of 10 consecutive loading cycles.

### 6.1. Influence of Waveform on Clamping Force

**Figure 9:**
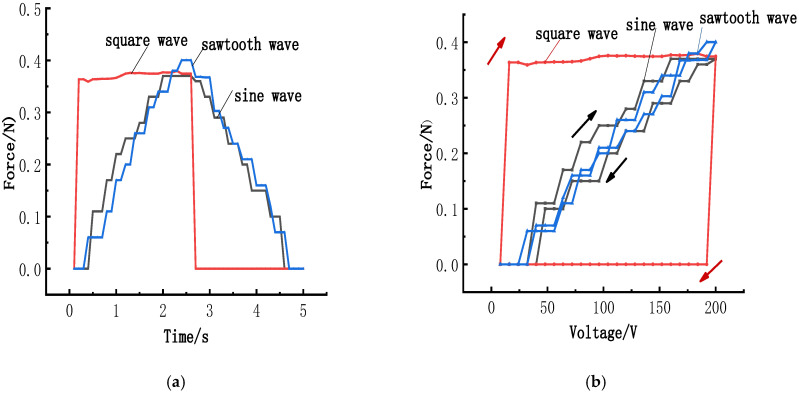
Influence of the electrical signal waveform on the radial force: (**a**) Force–time history versus the signals; (**b**) Force response versus the signals.

A piezoelectric actuator was subjected to square, sine, and sawtooth waveforms at 0.2 Hz and 200 volts to investigate the effect of different signal waveforms on its radial clamping force. The force–time history curves and the response curves for the radial clamping force are depicted in Figure 9.

The maximum radial force generated with a sawtooth waveform at a 50% time ratio is 0.40 N, compared to 0.37 N with a sine waveform, and 0.38 N with a square waveform, all at a constant frequency of 0.2 Hz and voltage of 200 V. As illustrated in Figure 9, the waveform of the electrical signal appears to have minimal impact on the maximum radial force of the piezoelectric elements. The radial force of the component does not vary significantly with changes in the electrical signal’s waveform. There is a smoother response of the clamping force under sine and sawtooth waveforms, while a faster response is observed under square waveforms.

### 6.2. Influence of the Voltage on the Clamping Force

**Figure 10:**
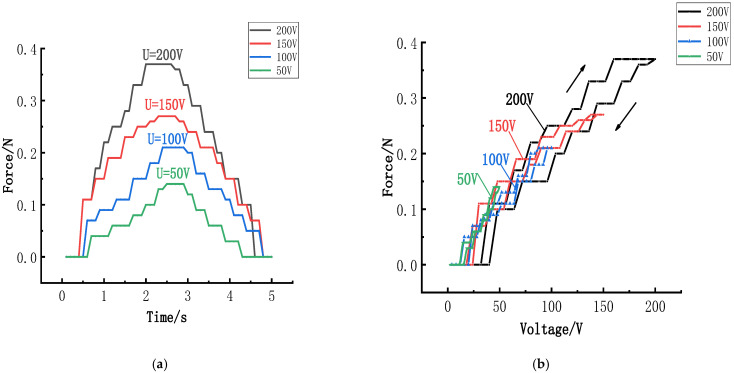
Influence of the voltage on the radial force: (**a**) Force–time history versus the voltage; (**b**) Force response versus the voltage.

Sawtooth signals are often used as electrical signals for stick–slip actuators due to their abrupt change in force response. Under square signals, the actuator exhibits a shock response mode, which could potentially damage the piezoelectric component if the test is prolonged. Consequently, sine waves, which feature relatively stable waveform changes, are selected as the excitation signal [38]. Piezoelectric specimens that require measurement are excited by a sine wave signal at a frequency of 0.2 Hz. Concurrently, the electrical signal was adjusted to 50 V, 100 V, 150 V, and 200 V, respectively, for testing purposes. Figure 10 displays the force–time history curves and response curves obtained.

A positive correlation can be observed between the radial clamping force of the piezoelectric actuator and the applied voltage. As the voltage decreases, the radial force correspondingly diminishes. This reduction in voltage decreases the number of electric domain flips within the piezoelectric sample. When an external electrical signal of 200 V is applied to the piezoelectric actuator, the radial force reaches 0.37 N. However, the force of the actuator decreases significantly to 0.14 N when the voltage is reduced to 50 V, resulting in a notable change in radial actuation force of 0.23 N. For external loads, the force exerted by the actuator is directly determined by the magnitude of the applied voltage.

### 6.3. Influence of the Frequency on Clamping Force

**Figure 11:**
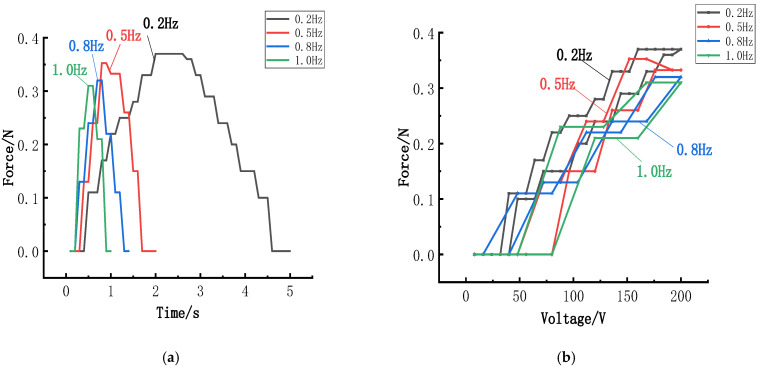
Influence of the frequency on the radial force: (**a**) Force–time history versus the frequency of the electrical signal; (**b**) Force response versus the frequency of the electrical signal.

Frequency is also an important parameter for the electrical signal. To investigate the relationship between the radial force generated by the piezoelectric actuator and the frequency of the electrical signal, piezoelectric actuators were excited with sine waves at 200 V. By varying the frequency of the electrical signal, the radial force versus time history curve and the corresponding response curve were obtained. The relationship between clamping force and frequency is depicted in Figure 11.

Piezoelectric actuators are operated in quasi-static states, where their operating frequency remains below their resonant frequency. In such conditions, the peak radial force of the actuator decreases as the frequency of the electrical signal increases, indicating a negative but weak correlation. Specifically, the increase in electrical signal frequency from 0.2 Hz to 1.0 Hz results in only a 0.06 N difference in peak force. This minor change illustrates that frequency variations have little impact on the radial force. However, the frequency of the signal can influence the response period of the radial force in the piezoelectric component. Operating at a lower signal frequency allows for the collection of more data points, providing a more accurate depiction of the radial force over time.

Experimental tests demonstrate that a piezoelectric actuator’s radial force is primarily influenced by changes in voltage, whereas variations in the electrical signal frequency have a minimal impact on the radial forces generated. Consequently, the radial force is adjusted by altering the voltage signal of the piezoelectric actuator, which, in turn, controls the deflection of the microgripper it drives [16]. This adaptation facilitates the application of micro-operations in the real micro-world. Additionally, piezoelectric actuators with interdigitated spiral electrodes generate a radial force on the disk when the circular edges are clamped. This radial force is utilized to generate the pumping action in MEMS-style micropumps, microvalves, and micromirrors [39,40]. The primary advantages of this actuator are its high micro-/nano-scale displacement, substantial force generation, and micro-/nano-second-range response, making it superior to other smart actuators.

## 7. Conclusions

In this study, a piezoelectric actuator with interdigitated spiral electrodes was analyzed for its output force characteristics. The formula representing the output actuation force in the radial direction was derived from the piezoelectric constitutive equation and the bond matrix transformation relationship. Finite element analysis was employed to examine the internal stress distribution of the piezoelectric actuator under clamping conditions. The analysis revealed a stress concentration at the non-zero potential end of the spiral electrode line. Meanwhile, to measure the radial output force of a piezoelectric actuator, several samples were prepared, and an experimental device for measuring clamping force was set up. Using this platform, the radial output force of the actuator was measured, and the influence of varying electrical signal parameters on the actuator’s output force was investigated. The results show that the waveform and frequency of the electrical signal do not significantly affect the radial force of the piezoelectric element. Instead, the applied voltage is the primary determinant of its radial force. An actuator equipped with interdigitated spiral electrodes, operated at 200 V and 0.2 Hz, generated a peak force of 0.37 N, which was 1.76 times greater than that produced by a previously utilized piezoelectric disc with conventional electrode structures. By symmetrically arranging spiral electrode lines on both sides of the piezoelectric disc, a radial electric field was created, enhancing the radial output force of the actuator through the joint coupling of the piezoelectric constants *d*_31_ and the much stronger *d*_33_. Moreover, the unique structure of the spiral electrode lines allows for more pairs of positive and negative electrode lines to be accommodated on the same limited circular surface compared to other interdigital piezoelectric disc actuators. This increases the active area affected by the piezoelectric effect within the ceramic disc, thereby enhancing the radial output force of the actuator. These findings provide a reference for the application of radial force output from disc-shaped piezoelectric ceramic actuators with interdigitated spiral electrodes.

## Figures and Tables

**Table 1:** Parameters of piezoelectric ceramic PZT-52.

Physical Symbols	PZT-52	Units
*d*_33_	575	10^−12^ C/N
*d*_31_	−260	10^−12^ C/N
*d*_15_	950	10^−12^ C/N
*ρ*	7600	kg/m^3^
*ε*_11_ *^T^*	3500	-
*ε*_33_ *^T^*	3250	-
*S*_11_ *^E^*	15.5	10^−12^ m^2^/N
*S*_12_ *^E^*	5.58	10^−12^ m^2^/N
*S*_13_ *^E^*	9.3	10^−12^ m^2^/N
*S*_33_ *^E^*	19	10^−12^ m^2^/N
*S*_55_ *^E^*	23	10^−12^ m^2^/N

## Data Availability

Data are available upon request from the corresponding author.
